# Editorial: Nutritional support in people living with human immunodeficiency virus (HIV)

**DOI:** 10.3389/fnut.2026.1779043

**Published:** 2026-01-29

**Authors:** Kaneez Fatima Jawwad

**Affiliations:** Department of Environmental Design, Health & Nutrional Sciences, Allama Iqbal Open University, Islamabad, Pakistan

**Keywords:** HIV, gut microbiota, chronic inflammation, food security, medical nutrition therapy (MNT), nutritional support, nutraceuticals

Frontiers in Nutrition aims to advance understanding of how nutrition influences health and disease across diverse populations and settings, with a strong emphasis on translational relevance, equity, and global impact. In the context of HIV, remarkable progress in antiretroviral therapy (ART) has reshaped the clinical trajectory of infection, transforming HIV into a chronic, manageable condition for millions worldwide. This success, however, has also underscored the need to address long-term health, functional capacity, and quality of life beyond viral suppression alone.

The widespread availability of ART has enabled many people living with HIV (PLHIV) to achieve near-normal life expectancy. As survival improves, increasing attention has shifted toward persistent immune activation, chronic inflammation, metabolic disturbances, neurocognitive impairment, and ongoing social and nutritional vulnerability. These challenges highlight that biomedical treatment, while essential, must be complemented by holistic approaches among which nutritional support remains a central yet still under-integrated component of comprehensive HIV care.

This Research Topic, *Nutritional Support for People Living With HIV*, brings together four complementary manuscripts examining nutrition across biological, clinical, and structural dimensions, spanning diverse geographical settings and age groups. Rather than approaching nutrition as a single intervention, the contributions collectively highlight its multifaceted role in shaping health outcomes, resilience, and quality of life among PLHIV.

## Contributions of the published manuscripts

At the population level, Boneya et al. present the multi-facility cross-sectional study “*Fruits and vegetables dietary intake and its estimated consumption among adults receiving antiretroviral therapy in health facilities in Northcentral Ethiopia*.” The study documents markedly low fruit and vegetable intake among adults receiving ART, falling well below recommended levels despite residence in agriculturally productive areas. These findings reveal critical gaps between food availability, dietary practices, and access to comprehensive nutritional education and counseling, emphasizing the need for context-specific dietary guidance integrated into routine HIV care.

Extending beyond individual dietary behavior, George et al. explore structural and policy influences in the qualitative *study* “*Understanding perspectives of HIV/AIDS affected households on food and nutrition interventions and social protection programmes in Zimbabwe*.” Drawing on lived experiences of HIV-affected households, the study illustrates how food insecurity, livelihood instability, and social protection mechanisms shape nutritional resilience. Importantly, the authors highlight the need for policy-driven program designs that explicitly define PLHIV as a vulnerable group and embed capacity building and resilience strengthening as core components to ensure sustainability.

Addressing pediatric vulnerability in humanitarian contexts, Abraham et al. report findings from an institution-based cross-sectional study titled “*Nearly half of HIV-positive children attending public health facilities are suffering from chronic under-nutrition in conflict-affected zones of Southern Ethiopia*.” The study reveals a substantial burden of chronic undernutrition among children receiving ART, underscoring the compounded effects of conflict exposure, recurrent infections particularly diarrhea limited dietary diversity, and household-level constraints. These findings emphasize the urgent need for early detection, continuous nutritional monitoring, and implementation of nutrition-sensitive programs in conflict-affected settings, with particular attention to larger households consuming limited food groups.

At the biological and mechanistic level, Dong et al. present a randomized controlled trial entitled “*Effects of Docosahexanoic Acid on Gut Microbiota and Fecal Metabolites in HIV-Infected Patients With Neurocognitive Impairment: A 6-Month Randomized, Double-Blind, Placebo-Controlled Trial*,” The study demonstrates that DHA algae oil supplementation favorably modulates gut microbiota composition, fecal metabolomic profiles, and markers of inflammation and oxidative stress among PLHIV with neurocognitive impairment. Although global cognitive scores did not significantly change, the observed biological shifts provide important insight into the gut–immune–brain axis as a pathway through which targeted nutritional interventions may support long-term prognosis.

## Biological pathways linking nutrition and HIV outcomes

Across the manuscripts, several interconnected biological pathways emerge as central to nutritional support in HIV care. These include gut microbiota alterations associated with HIV infection and long-term ART exposure, persistent chronic inflammation despite viral suppression, immune function sensitive to nutritional status, and metabolic stability influenced by prolonged ART use. Neurocognitive health further emerges as a critical domain through which nutrition may shape functional outcomes and overall quality of life.

## From current evidence to future priorities

While these studies provide valuable insights, they also highlight important gaps. Dietary patterns characterized by anti-inflammatory and antioxidant properties remain underrepresented in HIV-specific trials. Targeted nutraceuticals including DHA, omega-3 fatty acids, spirulina, and probiotics show promise as adjuncts to ART; however, larger and more diverse trials are required to clarify their clinical relevance across populations and settings.

Equally important is the delivery of nutrition through structured medical nutrition therapy (MNT) models that incorporate individualized assessment, counseling, and longitudinal follow-up. Lifestyle-related factors such as physical activity, sleep quality, and stress management further complement nutritional strategies and align with holistic models of chronic HIV care.

## An integrated framework for nutritional support in HIV

**Figure 1:**
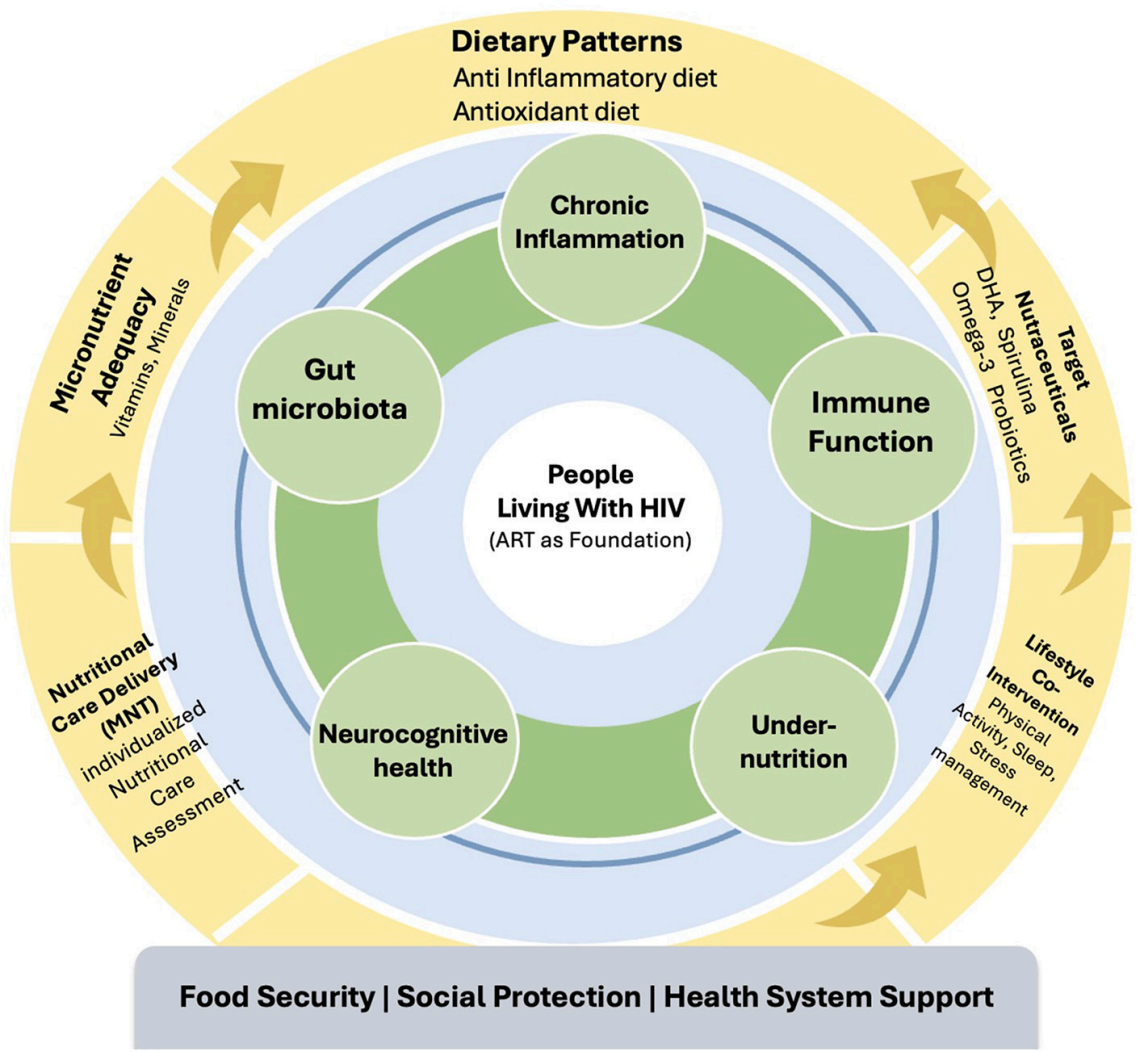
Integrated nutritional framework for HIV care. This figure synthesizes contributions of the published manuscripts by illustrating how nutritional support for people living with HIV operates across interconnected biological, interventional, and structural levels. The inner ring represents key biological pathways affected in HIV, including gut microbiota, chronic inflammation, immune function, metabolic stability, and neurocognitive health. The outer ring depicts nutritional and lifestyle strategies through which these pathways may be modulated, alongside antiretroviral therapy (ART), within a broader context of food security, social protection, and health system support.

These conceptual relationships are synthesized in Figure 1, which integrates the biological domains addressed by the included manuscripts (inner ring) with nutritional and lifestyle strategies representing both current applications and future priorities (outer ring). At the center of this framework are people living with HIV, with ART as the foundation of care. Nutritional strategies function as adjuncts to enhance long-term health and must be supported by equitable food systems, social protection mechanisms, and robust health infrastructure.

## Conclusion

Together, the four manuscripts included in this Research Topic provide a coherent and forward-looking perspective on nutritional support in HIV. By integrating biological, clinical, and structural insights, this Research Topic advances current understanding while identifying priorities for research, clinical practice, and policy development. Moving beyond viral suppression alone to address persistent nutritional challenges is essential for strengthening immune health, resilience, and long-term quality of life among people living with HIV.

